# Wastewater-Based Surveillance of Antibiotic Resistance Genes Associated with Tuberculosis Treatment Regimen in KwaZulu Natal, South Africa

**DOI:** 10.3390/antibiotics10111362

**Published:** 2021-11-08

**Authors:** Hlengiwe N. Mtetwa, Isaac D. Amoah, Sheena Kumari, Faizal Bux, Poovendhree Reddy

**Affiliations:** 1 Department of Community Health Studies, Faculty of Health Sciences, Durban University of Technology, P.O. Box 1334, Durban 4000, South Africa; 21303098@dut4life.ac.za; 2 Institute for Water and Wastewater Technology (IWWT), Durban University of Technology, P.O. Box 1334, Durban 4000, South Africa; Isaaca@dut.ac.za (I.D.A.); SheenaK1@dut.ac.za (S.K.); faizalb@dut.ac.za (F.B.)

**Keywords:** multidrug-resistant tuberculosis, antibiotic resistant genes, wastewater, droplet digital PCR, wastewater-based epidemiology

## Abstract

Essential components of public health include strengthening the surveillance of infectious diseases and developing early detection and prevention policies. This is particularly important for drug-resistant tuberculosis (DR-TB), which can be explored by using wastewater-based surveillance. This study aimed to use molecular techniques to determine the occurrence and concentration of antibiotic-resistance genes (ARGs) associated with tuberculosis (TB) resistance in untreated and treated wastewater. Raw/untreated and treated (post-chlorination) wastewater samples were taken from three wastewater treatment plants (WWTPs) in South Africa. The ARGs were selected to target drugs used for first- and second-line TB treatment. Both conventional polymerase chain reaction (PCR) and the more advanced droplet digital PCR (ddPCR) were evaluated as surveillance strategies to determine the distribution and concentration of the selected ARGs. The most abundant ARG in the untreated wastewater was the *rrs* gene, associated with resistance to the aminoglycosides, specifically streptomycin, with median concentration ranges of 4.69–5.19 log copies/mL. In contrast, *pncA* gene, associated with resistance to the TB drug pyrazinamide, was the least detected (1.59 to 2.27 log copies/mL). Resistance genes associated with bedaquiline was detected, which is a significant finding because this is a new drug introduced in South Africa for the treatment of multi-drug resistant TB. This study, therefore, establishes the potential of molecular surveillance of wastewater for monitoring antibiotic resistance to TB treatment in communities.

## 1. Introduction

Antimicrobial resistance (AMR), a major threat to global health and a growing concern worldwide [1,2], is mainly attributed to the excessive use and misuse of antibiotics [3]. AMR related to tuberculosis (TB) has been extensively reported in clinical studies [4,5,6]. South Africa has one of the highest recorded prevalence of drug-resistant tuberculosis (DR-TB) patients globally [7,8] and particularly with the increasing burden of human immunodefiency virus (HIV) coinfection [9]. Drug-susceptible TB and DR-TB treatment regimen are classified into two groups: first-line and second-line TB drugs [10,11]. The TB/DR-TB treatment regimen in South Africa includes first-line TB drugs such as isoniazid, rifampicin, ethambutol and pyrazinamide, while the second-line TB drugs used for rifampicin-resistant (RR-TB), multidrug-resistant (MDR-TB) and extensively drug-resistant tuberculosis (XDR-TB) include ofloxacin, moxifloxacin, bedaquiline, ethionamide, kanamycin and amikacin [12]. Other drugs, such as streptomycin, have been reported in both first-line and second-line treatment regimens [13]. The One Health approach to combating AMR argues that human, animal and environmental health are interconnected and all sectors should be considered in the fight against AMR [14]. AMR in environmental matrices, such as wastewater, surface water and treated water, has been reported in various studies [15,16,17]; however, none of these studies focused specifically on tuberculosis resistance or related genes in wastewater. The presence of these genes in wastewater may be attributed to secretions in feces and urine of infected individuals or animals. In urban areas with centralized sewage systems, the wastewaters discharged from households, hospitals and pharmaceutical industries are collected and treated together in wastewater treatment facilities [18,19], and this wastewater reflects the health and habits of the community served by that treatment plant [20]. Analyses of untreated wastewater (influent) could therefore provide an insight into the prevalence of drug-resistant TB in the served population. The analysis of raw wastewater for surveillance of infections in connected populations has been proposed by several researchers [20,21,22,23]. This approach, referred to as wastewater-based epidemiology (WBE), could therefore contribute to the development of alternate AMR surveillance systems for TB to complement the existing clinical-based surveillance, hospital-admission data, questionnaires, surveys, motility and morbidity rates and sentinel surveillance systems [20]. Linking WBE and the One Health approach in monitoring and management of the occurrence and spread of drug-resistant TB in the population could contribute to early detection and public-health mitigation strategies.

Additionally, AMR development in wastewater could be due to the presence of antimicrobials in this environment. It is reported that only 30% of the antibiotics consumed are metabolized in the human body, whilst the major percentage is released to the environment through feces and urine, either in their original form or as residues and conjugates [9]. Therefore, the extensive use of antibiotics used in TB/DR-TB treatment regimens could result in the excess release of antibiotics in wastewater and related environments, especially where there is a high prevalence of TB in the population [24]. The presence of these antibiotics/antimicrobials, together with the high-bacterial-density, high-nutrient and high-oxygen conditions within wastewater treatment plants (WWTP) provides conducive conditions for the transfer of antibiotic resistance genes (ARGs), development of new antibiotic resistant bacteria (ARB) and for creating hotspots for the spread of resistant bacteria and genes in the environment [9,25]. Therefore, the detection of ARGs responsible for resistance to tuberculosis drugs in untreated and treated wastewater can be used to estimate the contribution of WWTPs in the dissemination of ARGs. However, it must be noted that this means of antimicrobial resistance may not be the main scenario for the development of antimicrobial resistance in *M. tuberculosis* due to their fastidious nature. Therefore, the detection of the ARGs responsible for antimicrobial resistance in tuberculosis infections in wastewater could be mainly due to the resistance in the connected communities.

This study presents the molecular surveillance of ARGs associated with drug resistance in tuberculosis infections in both treated and untreated wastewater from Kwa-Zulu Natal, South Africa. This surveillance, through the use of both conventional and droplet digital polymerase chain reaction (ddPCR) techniques, may advocate for the use of WBE for drug-resistant TB surveillance.

## 2. Materials and Methods

### 2.1. Study Site

Three WWTPs, receiving municipal wastewater in the city of Durban, South Africa, referred to as WWTP A, WWTP B and WWTP C, were investigated in this study. The selection of the WWTPs was based on plants serving at least a population of 10,000 individuals and those receiving hospital sewage. These WWTPs also had different treatment configurations and capacities, details of which are presented in Table 1. Wastewater samples were taken on three separate occasions.

### 2.2. Sample Collection and Processing

A one-liter composite sample was taken from the influent (raw/untreated wastewater) and final effluent (post chlorinated effluent) at each WWTP. Thus, two one-liter samples (influent and effluent) were taken per WWTP; many subsamples (100 mL) were taken every 30 min, until a final volume of 1 L was achieved.

Samples were transported to the laboratory in a cooler box with ice, stored at 4 °C and analyzed within 48 h. Before analysis, samples were homogenized, and 50 mL subsamples were taken and centrifuged at 3000 rpm for 20 min; the supernatant was discarded, and the total DNA was extracted from the pellet, using a DNeasy Powersoil DNA extraction kit (QIAGEN, Hilden, Germany), following the extraction procedure provided by the manufacturer, with no modification. The volumes of sample and pellets after centrifugation were noted to help with calculation of concentrations afterwards. The quantity and quality of the extracted DNA were determined using the IMPLEN NanoPhotometer NP80—All-in-One Spectrophotometer. All analyses were conducted in triplicates.

### 2.3. Selection of Antibiotic Resistance Genes

The genes responsible for resistance to a variety of antibiotics used in drug-susceptible and drug-resistant tuberculosis (TB/DR-TB) treatment regimens were selected for the study (Table 2), based on the availability of information and common usage in South Africa [27]. Those antibiotics used exclusively for treating TB infections are categorized into the first and second-line treatment regimen. The first-line treatment regimen drugs are isoniazid, rifampicin, ethambutol and pyrazinamide [28,29,30]. The second-line treatment regimen are kanamycin, amikacin, delamanid, bedaquiline and ethionamide [28,31]. Additionally, other genes coding for resistance to drugs that may be used to treat other infections apart from TB were included, as they are sometimes used as part of the TB treatment regimen. These are streptomycin, cycloserine, ofloxacin and moxifloxacin [28,32].

### 2.4. Optimization of Polymerase Chain Reaction Conditions

Method optimization was conducted by using published primers targeting regions of the selected gene presented in Supplementary Materials (Table S1) that have been reported to be responsible for the occurrence of antibiotic resistance. These primer sequences were initially verified by using the Basic Local Alignment Search Tool (BLAST) of the National Center for Biotechnology Information (NCBI BLAST) tool prior to synthesis and optimization. The PCR mixture for all targeted genes contained 12.5 μL of OneTaq 2X Master Mix with Standard Buffer (New England BioLabs Inc., Ipswich, MA, USA), 2 μL of primer mix containing 1 μL of each primer set, (final concentration of 0.2–0.4 µM), 1 μL (20 ng/µL) of DNA template and 9.5 μL of molecular grade water in a final volume of 25 μL in a reaction tube. PCR amplification was performed in a Veriti™ 96-Well Thermal Cycler. Optimized thermocycling conditions were initial denaturation step at 95 °C for 10 min and followed by 30 cycles of denaturation at 96 °C for 45 s. Annealing temperatures varied for each primer: *eis* (50 °C for 60 s), *gyrB*, *rrs*, *ethR* (52 °C for 60 s), *pncA*, *gyrA*, *katG*, *aptE* (54 °C for 60 s), *rpoB*, *embB* (60° C for 60 s) and extension at 72 °C for 40 s. The final extension step was performed at 72 °C for 10 min.

PCR products were analyzed by 2% agarose gel electrophoresis performed at 70 V in 1 X Tris-Borate EDTA (TBE) buffer. The agarose gel was pre-stained with ethidium bromide (final concentration of 0.2–0.5 μg/mL). The electrophoresed PCR products were visualized by using a Bio-Rad Gel Doc™ XR, a gel documentation system.

The optimized PCR protocol above was used to determine the presence of the selected genes coding for tuberculosis resistance in wastewater from Durban, South Africa. Wastewater samples were taken and processed by using the protocol described in Section 2.2.

### 2.5. Optimization of ddPCR for Detection and Quantification of Tuberculosis-Resistance Genes in Wastewater

Concentration of the selected ARGs in the wastewater samples was determined by using ddPCR analysis. The ddPCR was performed in a 22 μL reaction volume, containing 10 μL of 2X QX200 ddPCR EvaGreen Supermix (Bio-Rad, Hercules, CA, USA), 1 μL (20 ng/µL) of template DNA, 1.25 μL each of the forward primers (FP) and reverse primers (RP), each at a final concentration of 250 nM and RNase/DNase free water.

Droplets were generated by using the automated droplet generator (Bio-Rad) and were amplified by using a C1000 Touch™ Thermal Cycler (Bio-Rad). The following thermal cycling conditions were followed: initial denaturation step at 95 °C for 10 min, followed by 45 cycles of denaturation at 96 °C for 45 s. The annealing temperatures varied for each primer (genes): *eis* (50 °C for 60 s), *gyrB*, *rrs*, *ethR* (52 °C for 60 s), *pncA*, *gyrA*, *katG*, *aptE* (54 °C for 60 s), *rpoB*, *embB* (60 °C for 60 s). The incubation step was performed at 98 °C for 10 min (ramp rate 2.2 °C/s) and held at 4 °C for 30 min before reading the plate. After thermal cycling, the ddPCR plates were read, using the QX200 droplet reader (Bio-Rad). Droplet counts and amplitudes were exported to and analyzed with the QuantaSoft™ analysis Pro software (Bio-Rad). The potential impact of PCR inhibitors was determined in the protocol-development phase via 10-fold serial dilution of the DNA extracts and the concentrations determined with the ddPCR protocols. No PCR inhibition was observed based on the concentration of the ARGs measured.

### 2.6. Statistical Analysis

Descriptive statistics were performed with Microsoft Excel, and a test of normality was performed based on the Akaike Information Criterion (AIC) score measured with @Risk (Palisade Inc., Ithaca, NY, USA). Based on the normality tests, a comparison of the concentration of the different genes responsible for tuberculosis resistance and the three WWTPs was achieved by using the Kruskal–Wallis tests, followed by Dunn’s multiple comparison tests. The difference in the concentration of the genes between untreated and treated wastewater was compared using the Mann–Whitney tests. All data were log-transformed (log10 copies/mL) prior to analysis. All statistical tests were performed at a 95% confidence interval, and a *p*-value of ≤0.05 was considered statistically significant. All statistical analyses were performed using GraphPad Prism (Version 7.0, GraphPad Software, San Diego, CA, USA).

## 3. Results

### 3.1. Detection of Genes Associated with Resistance to Drugs Used in TB Treatment Regimen Using Conventional PCR

The genes associated with resistance to TB/DR-TB drugs used in this study were found in the majority of the samples tested from all the three WWTPS investigated. The genes coding resistance to the second-line TB drugs, such as moxifloxacin, bedaquiline, ethionamide and kanamycin/amikacin (*gyrB*, *atpE*, *ethR* and *eis*), were the most frequently detected from both influent and effluent samples (Table 3). However, the *gyrA* gene associated with fluoroquinolone resistance, specifically ofloxacin, used in the second-line treatment regimen, was only detected from one of the treatment plants (WWTP C) investigated.

**Figure 1:**
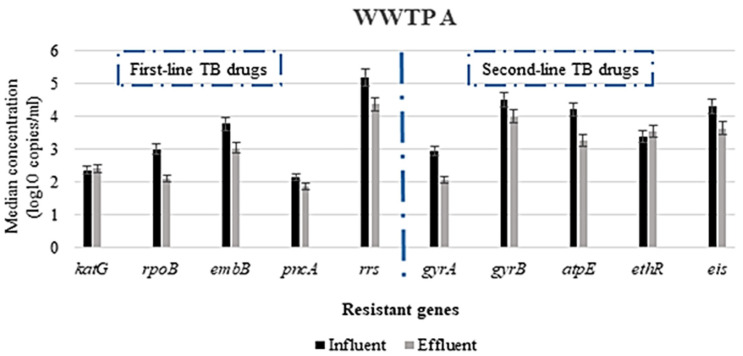
Median log copies/mL concentration of selected antimicrobial resistance genes in WWTP A.

**Figure 2:**
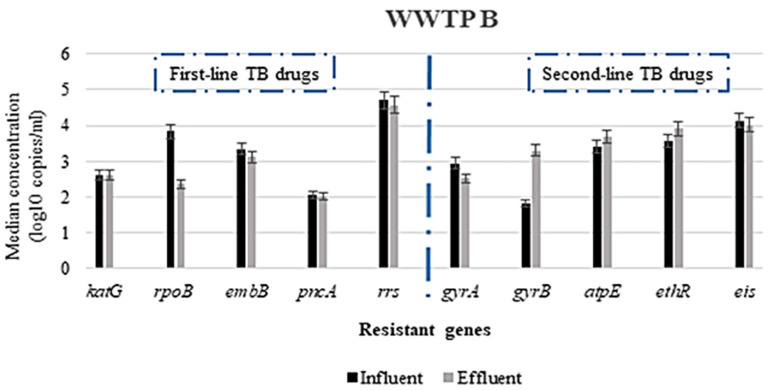
Median log copies/mL concentration of selected antimicrobial resistance genes in WWTP B.

**Figure 3:**
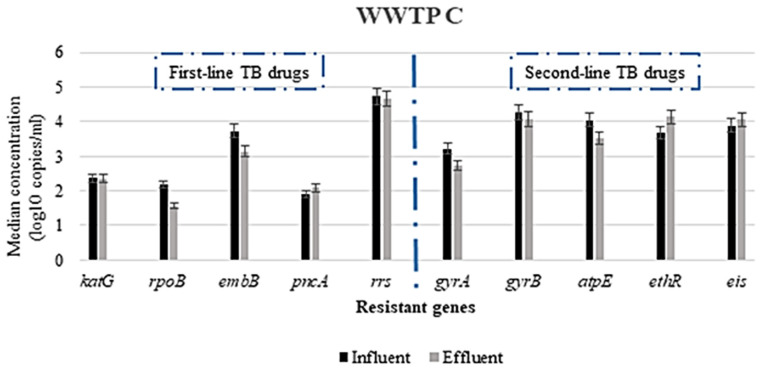
Median log copies/mL concentration of selected antimicrobial resistance genes in WWTP C.

The prevalence of the genes responsible for resistance to first-line TB drugs varied depending on the gene, WWTP and the type of PCR analysis used. All the wastewater samples analyzed were positive for all the genes investigated, using ddPCR for the detection; however, there were some genes not detected via conventional PCR. The *rrs* gene (conferring resistance to streptomycin) was found in all wastewater samples, both influent and effluent. In contrast, the gene associated with the resistance to pyrazinamide (*pncA*) was not detected in any of the samples via conventional PCR. However, the *pncA* gene was detected in all wastewater samples, albeit in low concentrations (Figure 1, Figure 2 and Figure 3), using the ddPCR. Other less frequently detected ARGs included the *rpoB* gene associated with rifampicin resistance. Table 3 presents the prevalence of the various ARGs in wastewater from all the WWTPs.

### 3.2. Abundance of Antimicrobial Resistance Genes in Untreated and Treated Wastewater

#### 3.2.1. Concentration of ARGs in the WWTPs

The highest concentration of ARGs in both influent and effluent in WWTP A was the *rrs* gene, conferring resistance to streptomycin, with concentrations ranging from 4.35 to 5.19 log copies/mL. The other most abundant ARGs in both influent and effluent wastewater were *gyrB*, *eis*, *aptE*, *embB* and *ethR* genes associated with resistance to fluoroquinolones, (specifically moxifloxacin), aminoglycosides (kanamycin/amikacin), bedaquiline, ethambutol and ethionamide, respectively, with median concentrations ranging from 3.05(±0.08) to 4.51(±0.04) log copies/mL. As shown in Figure 1, the least abundant ARG among the genes was *pncA*, with measured concentrations ranging from 2.10 to 2.20 log copies/mL in the influent and from 1.80 to 1.96 log copies/mL in the effluent. However, no statistically significant difference was observed when the influent and effluent concentrations were compared using the Mann–Whitney test at a 95% confidence interval. A similar trend was observed in WWTP B (Figure 2) and WWTP C (Figure 3), where the highest concentration of ARGs in both influent and effluent was the *rrs* gene, and the least abundant ARG was the *pncA* gene.

These results show that the ARG with the highest concentration in all the WWTPs was the *rrs* gene; however, there was no statistically significant difference (*p* ≥ 0.05) in the concentrations of this gene in the influent samples of all WWTPs when compared. In contrast, a significant difference in their concentration was observed in the effluent samples when all the WWTPs were compared (*p* ≤ 0.05). In addition to *rrs*, the other ARGs found abundantly across all the WWTPs were the *eis*, *ethR* and *atpE* genes with median concentrations ranging from 3.27(±0.03) to 4.30(±0.09) log copies/mL for both influent and effluent samples. Irrespective of the WWTP, the least concentration of ARGs detected was the *pncA* gene, with no statistically significant difference in the concentrations when all WWTPs were compared (*p* ≥ 0.05). Furthermore, no statistically significant difference (*p* ≥ 0.05) was achieved when comparing the influent to effluent concentrations of all the ARGs within each WWTP and across all the WWTPs, using the Mann–Whitney test at a 95% confidence interval.

#### 3.2.2. Variation in the Concentration of ARGs Responsible for Resistance to First- and Second-Line TB-Treatment Drugs and Other Related Drugs

Comparatively, the difference in concentration of the ARGs associated with resistance to first- and second-line TB treatment drugs in all the WWTPs was statistically significant (*p*-value ≤ 0.05), with the exception of the concentrations of *gyrA*. The statistical difference in the *eis* and *ethR* concentration was driven by differences between WWTP A and WWTP C, and the difference in the *atpE* and *gyrB* concentrations was driven by differences between WWTP A and WWTP B. However, both the Kruskal–Wallis test and Dunn’s multiple comparison tests did not show any statistically significant differences in the concentration of *gyrA* between the various WWTPs (*p*-value ≥ 0.05).

### 3.3. Reduction in Antimicrobial Resistance Genes during Wastewater Treatment

**Figure 4:**
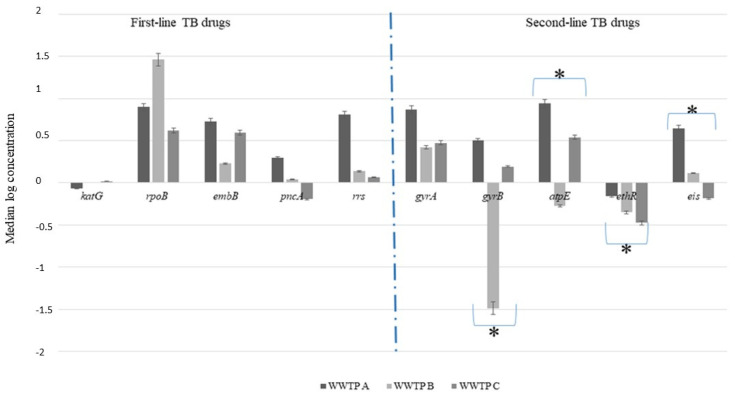
Median (SD) log reduction achieved for the various antimicrobial-resistance genes in the three wastewater treatment plants. * The difference in median log reduction for the ARGs marked was statistically significant, using the Kruskal–Wallis tests (*p*-value ≤ 0.05).

The concentration of the various ARGs detected differed between the untreated and treated wastewater samples (Figure 1, Figure 2 and Figure 3). In most instances, the concentrations in the treated wastewater (effluent) were higher than in the untreated wastewater. For instance, the highest log reduction in these ARGs was observed for *rpoB*, *gyrA* and *embB*, with median log reduction ranges of 0.62–0.89, 0.41–0. 87 and 0.23–0.73, respectively (Figure 4). In contrast, for some of the ARGs, the concentrations measured in the treated wastewater were higher than in the untreated wastewater. This was observed for *ethR* in all the WWTPs, with median log reductions ranging from −0.16 to −0.47. In WWTP B, an increase of effluent concentrations for *gyrB* and *atpE* was observed, where *gyrB* median log reduction was −1.49 and −0.27 for *atpE* (Figure 4). These results show that the concentration of these ARGs in the treated wastewater was still high, ranging from 1 to over 4 log copies/mL, as shown in Figure 1, Figure 2 and Figure 3.

In most instances, the log reduction of ARGs associated with first-line TB treatment did not show any statistically significant differences between all three WWTPs (*p*-value ≥ 0.05). In contrast, all ARGs associated with second-line TB treatment (except *gyrA*), as well as all the other drugs used in TB treatment, showed statistically significant differences in the log reduction between all WWTPs (*p*-value ≤ 0.05). The statistically significant differences observed in log reductions achieved for *atpE* and *gyrB* genes was due to the differences between WWTP A and WWTP B. The difference in the log reductions between WWTP A and WWTP C was responsible for the statistically significant reduction reported for the *eis* and *ethR* genes. When comparing log reductions between first-line and second-line ARGs, no statistically significant difference was observed at a 95% confidence interval, using the Mann–Whitney test.

## 4. Discussion

Antibiotic resistant bacteria (ARB) and genes (ARGs) are an emerging concern [38] to public health worldwide, and DR-TB is a global health concern that threatens the significant progress made in TB care and prevention. South Africa is one of the countries with the highest prevalence of drug-resistant tuberculosis (DR-TB) globally [7,8]. Therefore, there is a need for alternative surveillance systems to augment the fight against DR-TB. This study successfully optimized both conventional and droplet digital PCR assays for the detection of ARGs associated with TB treatment, and therefore showing the potential of using wastewater as a snapshot of ARG circulation in the connected population. However, the utility of this approach is largely dependent on the sensitivity of the methods used. The use of two PCR assays with different sensitivities in this study highlighted the importance of these sensitive assays in wastewater-based surveillance for ARGs. Using conventional PCR, we found that ARGs responsible for resistance to the second-line TB treatments were present in all samples; in contrast, the prevalence of ARGs responsible for resistance to first-line TB drugs was lower. Based on conventional PCR analysis, *pncA* was not detected in any of the samples, and there was a low frequency of detection for *rpoB*. However, all the genes were detected by using ddPCR. This could be due to the sensitivity of the ddPCR platform to detect even the lowest concentrations compared to other PCR platforms [39,40,41]. The concentrations of these ARGs as determined with ddPCR provided a better estimation of occurrence in wastewater, providing an indication of the prevalence of these ARGs in the populations served by the different WWTPs. However, it must be noted that ddPCR is not readily available in several laboratories, due to the high costs associated with its use and the need for highly skilled personnel. This may be a limitation for the adoption of this technology for routine analysis.

The most abundant ARG was *rrs*, which is responsible for streptomycin resistance [35,42]. The high concentration of this ARG could be due to the common use of streptomycin, not just for both drug-susceptible and drug-resistant TB infections but also for the treatment of other infections caused by other *Mycobacterium*-related species [43] and other bacteria, such as *Xanthomonas* spp. [38], *Salmonella typhimurium* and *Yersinia pestis* [44]. Detection of the *rrs* gene responsible for streptomycin resistance in clinical *M. tuberculosis* isolates has been reported in South Africa [45], China [46], Iran [47] and Myanmar [48]. Additionally, the *rrs* gene has been implicated in cross-resistance to other aminoglycosides (amikacin/kanamycin) that are also used in TB treatment [15]. Therefore, the high prevalence and concentration of this gene could be attributed to the high prevalence of resistance to streptomycin or the aminoglycosides in the connected populations or prevalence of this gene in microorganisms in the wastewater environment.

In contrast, the *pncA* gene concentrations were very low in the wastewater across all WWTPs, and this could explain the non-detection of this gene by the conventional PCR. This gene (*pncA*) confers resistance to pyrazinamide, which is used exclusively for the treatment of active TB (not latent TB) [35]. This drug is mostly used in combination with rifampicin, isoniazid and either streptomycin or ethambutol [49,50]. This shows that DR-TB may be driven by resistance to rifampicin or isoniazid and streptomycin or ethambutol, while pyrazinamide remains relatively effective [51]. However, Allana et al. [52] reported that 70% of multidrug-resistant and 96% of extensively drug-resistant *M. tuberculosis* isolates in South Africa and Georgia were positive for the *pncA* gene. Detection of this gene in clinical *M. tuberculosis* isolates has been published from other countries, such as Pakistan [53] and Uganda [54]. The data obtained in our study, therefore, show that the environmental abundance of this gene could be driven by bacteria carrying this resistance gene in the wastewater environment or a prevalence within the local population/communities.

A major finding worth highlighting in this study is the abundance of the ARG *aptE,* which is responsible for resistance to bedaquiline. Bedaquiline is one of the newer drugs added to the MDR-TB treatment regimen in South Africa that has been associated with a reduced DR-TB mortality rate [55]. This antibiotic is used as either add on to existing treatment regimens or used in cases of resistance, to certain core TB/MDR-TB drugs. The abundance of this ARG may allude to the existence of resistance to this antibiotic already in South Africa, either circulating in the connected human population or among bacteria in wastewater. This result therefore shows a potential high prevalence of MDR-TB which may contribute to the epidemiological profile of the population served by these WWTPs.

The relatively high concentration of these ARGs may not only be associated with TB treatment or even mycobacteria infections. For instance, the *rpoB* gene, which is responsible for resistance to rifampicin, has been reported in environmental mycobacteria, such as *Mycobacterium abscessus* [25,29], and other bacteria, such as *Staphylococcus aureus* [28,29], *Streptococcus* spp. [5], *E. coli* [29] and other species [29]. Additionally, fluoroquinolone-resistance genes (*gyrA*/*gyrB*) have also been reported in *Legionella pneumophila* [56]. The detection of these ARGs may be attributed to several other factors, such as high concentrations in wastewater (due to improper waste management) of unused/expired drugs and overuse of TB/MDR-TB medication. The presence of the antibiotics, together with the high bacterial density, high nutrient and high oxygen conditions within WWTPs, provides conducive conditions for the transfer of ARGs, development of new ARB and for creating hotspots for the spread of resistant bacteria and genes into the environment [9,25]. This may not necessarily relate to the presence of antibiotics in higher concentrations in the wastewater, as ARBs and ARGs have been reported in the environment without the actual presence of antibiotics relating to resistance [17,57]. Horizontal gene transfer (HGT) is also reported as the most common route for which the ARGs are transferred among different bacterial communities in the WWTPs, which could also account for the higher concentrations in the effluent samples found in our results. Similar findings have been reported by other authors [51,57,58].

The reduction in the concentration of the ARGs associated with first-line TB treatment did not show any statistically significant differences (*p*-value ≥ 0.05). This could be a result of numerous reasons, including the ineffectiveness of the treatment plants in removing these ARBs and ARGs or the ability of these ARBs to survive and attach to sludge due to their hydrophobic nature [57,59,60,61] or genes being carried by more than one type of bacteria [62]. The negative log reductions (or increased concentrations in the effluent) observed for the genes responsible for resistance to second-line drugs raise concerns due to the release of high concentrations (Figure 1, Figure 2 and Figure 3) of these ARGs into surface water, which could be attributed to the presence of *Mycobacterium* spp. in high concentrations in treated wastewater [63] or cross-resistance in the bacterial communities in wastewater. The high concentrations in the treated wastewater, sometimes higher than the untreated wastewater, could be due to selective pressures that increase the concentrations of ARB by inhibiting antibiotic susceptible bacteria or contribute to the selection of mutations [64]. Additionally, cell lysis/disruption from different chemical- and physical-treatment processes used in the wastewater treatment [65,66], leading to the release of the nucleic material in the wastewater, could also account for the high concentration observed in the treated wastewater. Some of the treatment processes, such as biofiltration, conventional activated sludge and biological nutrient removal, have been reported to promote the growth of slow-growing microbial populations in wastewater due to higher solids-retention time or sludge recirculation [67,68]. This could also explain the high concentrations of the *rrs*, *ethR* and *atpE* genes found in the effluent of especially WWTP B for the ARGs when all WWTPs were compared. This WWTP is the only one with an activated sludge process incorporating an extended aeration treatment. Therefore, the increase in concentration of the ARGs during treatment in this WWTP could potentially be mediated by this treatment process. Additionally, protozoans have been reported as environmental hosts for *Mycobacterium* spp. They are known to survive within protozoa [6,69]. This could be one means of survival for this bacterium within the treatment plants and also could bypass the disinfection stage, as protozoans are resistant to chlorination. However, these results are inconclusive to categorically state the impact of the treatment process on removal of the ARGs. These ARGs could either be present in dead bacterial cells, live/viable bacteria or even extracellular. Therefore, there is the need for further studies in this regard, especially taking into consideration the potential health implication and risk associated with the release of ARBs and ARGs in treated wastewater.

## 5. Conclusions

Wastewater-based surveillance of antibiotic resistance associated with TB treatment that uses advanced molecular techniques provides a unique opportunity to complement surveillance systems aimed at monitoring TB resistance. The results obtained in this study show the potential of wastewater analysis in providing an insight into antimicrobial resistance. This study was able to show that resistance to drugs used in both first- and second-line TB treatment is common in untreated wastewater. This could be due to widespread circulation in the populations or abundance of bacteria carrying these genes in the wastewater environment. Furthermore, this study shows that the concentration of these ARGs is associated with the extent of use of the drugs in TB treatment, due to the observation that antibiotics used in TB treatment and other infections, such as streptomycin, had a higher prevalence and concentrations compared to lesser used drugs, such pyrazinamide. This, therefore, shows the potential of wastewater-based surveillance systems in complementing the existing TB-resistance surveillance systems.

This study also established that wastewater treatment plants have varying efficiencies in removing these ARGs. However, there was no distinct WWTP configuration that was observed to have higher efficiency in removing these ARGs. Concentrations of up to 4 log copies/mL of these ARGs were still detected in the treated wastewater, which could potentially result in the spread of these resistance genes in the aquatic environment. These genes could be extracellular or carried in the mycobacterial cell or other bacterial species. Therefore, further studies are recommended to ascertain whether the genes detected in the treated wastewater are either extracellular or carried in viable microorganisms and to understand the diversity of these carrier bacteria. Furthermore, future studies could also include identifying the potential bacterial communities carrying these genes through advanced microbial community analysis. Additionally, the WWTP operational parameters could be included to ascertain their potential impact on ARG removal, although this was not observed in the current study.

## Figures and Tables

**Table 1:** Details of the wastewater treatment plants used for this study.

Treatment Works	Design Capacity (Mℓ/d)	Hydraulic Retention Time (h) *	Primary Settling	Activated Sludge	Secondary Clarification	Bio-Filters	Sludge Digestion	Tertiary Treatment	Remarks
WWTP A	18.8	31	Y	N	N	Y	Y	Y	Receives from wastewater from a hospital that offers health services to the community at regional and district levels and has 17 clinics attached. This hospital is one of the sites for Mother-to-Child-Transmission (MTCT) of HIV and has the largest crisis centre.
WWTP B	4.90	36	N/A	Extended Aeration	Y	N	N	Y	Receives from a hospital that serves predominantly residential areas. This is also a referral hospital for another hospital and clinic
WWTP C	70.0	38	Y	Conventional	Y	N	Y	Y	Receives from a hospital complex which offers specialized services for Multidrug-resistant (MDR) and complicated TB

Availability of the treatment process (Y = yes/N = no), Information sourced from Reference [26]. * Calculated HRT.

**Table 2:** Antibiotic resistance genes selected for this study and the antibiotics that they code resistance to.

	Chemical Class	Drug Name	Gene	References
**First-line drugs**	Pyridine	Isoniazid (H)	*katG*	[11,33,34,35,36]
	Rifamycin	Rifampicin (R)	*rpoB*	
	Ethylenediamine	Ethambutol (E)	*embB*	
	Pyrazine	Pyrazinamide (Z)	*pncA*	
Add-on drug	Aminoglycoside	Streptomycin (S)	*rrs*	
**Second-line drugs**	Fluoroquinolone	Ofloxacin (Ofx)	*gyrA*	[11,34,37]
		Moxifloxacin (Mfx)	*gyrB*	
	Diarylquinoline	Bedaquiline (Bdq)	*atpE*	
	Pyridine	Ethionamide (Eto)	*ethR*	[11,33,34]
Injectable drugs	Aminoglycoside	Kanamycin (Km)	*eis*	[11,33,34]
		Amikacin (Amk)	*eis*	

**Table 3:** Detection of antimicrobial resistance genes associated with drug-resistant TB.

			Percentage (%) of Samples Positive (* *n* = 9 per Sampling Point)											
Category of Treatment	Gene Detected	Antibiotic/s	WWTP A				WWTP B				WWTP C			
			Conventional PCR		ddPCR		Conventional PCR		ddPCR		Conventional PCR		ddPCR	
			Influent	Effluent	Influent	Effluent	Influent	Effluent	Influent	Effluent	Influent	Effluent	Influent	Effluent
First-line TB treatment	*katG*	H	100	100	100	100	100	100	100	100	0	100	100	100
	*rpoB*	R	100	0	100	100	0	0	100	100	100	100	100	100
	*embB*	E	100	100	100	100	100	0	100	100	100	100	100	100
	*pncA*	Z	0	0	100	100	0	0	100	100	0	0	100	100
	*rrs*	S	100	100	100	100	100	100	100	100	100	100	100	100
Second-line TB treatment Other drugs	*gyrA*	Ofx	0	0	100	100	0	0	100	100	100	100	100	100
	*gyrB*	Mfx	100	100	100	100	100	100	100	100	100	100	100	100
	*atpE*	Bdq	100	100	100	100	100	100	100	100	100	100	100	100
	*ethR*	Eto	100	100	100	100	100	100	100	100	100	100	100	100
Injectables	*eis*	Km/Amk	100	100	100	100	100	100	100	100	100	100	100	100

* Samples were taken on three separate occasions for each WWTP, and analysis was performed in triplicate for each organism. H, isoniazid; R, rifampicin; E, ethambutol; Z, pyrazinamide; S, streptomycin; Ofx, ofloxacin; Mfx, moxifloxacin; Bdq, Bedaquiline; Eto, ethionamide; Km/Amk, kanamycin/amikacin.

## Data Availability

All the data generated is presented in the manuscript and the supplementary material.

## Supplementary Materials

The following are available online at https://www.mdpi.com/article/10.3390/antibiotics10111362/s1. Table S1: Target resistant genes and their PCR primer sequences. Table S2: Median (±SD) Log10 concentrations of the antimicrobial resistance genes measured in the influent and effluent wastewater. Figures S1–S3: Diagrams for the wastewater treatment plant’s configurations chosen for this study [70,71,72,73,74,75].
