# Supplementary material for: Wastewater-Based Surveillance of Antibiotic Resistance Genes Associated with Tuberculosis Treatment Regimen in KwaZulu Natal, South Africa
Source: Antibiotics (Basel). 2021 Nov 8;10(11):1362. doi: 10.3390/antibiotics10111362 (PMC8614817; doi:10.3390/antibiotics10111362)
Supplement: Supplementary file 1 [file antibiotics-10-01362-s001.zip › antibiotics-1349525-supplementary.pdf]

Table S1: Target resistant genes and their PCR primer sequences

**Target gene- locus**	**Annotation**	**Drug name**	**Primer sequence**	**References**
rpoB	DNA-directed RNA polymerase subunit beta	Rifampicin	F:5'-CGAGGTGCCGGTGGAAAC-3'	[71; 72; 24; 10]
			R:5'-GTCGTCGTGCTCCAGGAAGG-3'	
KatG	catalase-peroxidase	Isonizaid	F:5'-GAGCCCGATGAGGTCTATTG-3'	[71; 72; 24; 10]
			R:5'-GTCCTTGGCGGTGTATTGC-3'	
inhA	NADH-dependent enoyl-[ACP] reductase		F:5'-GAGCGTAACCCCAGTGCGAA-3'	[24; 10]
			R:5'-TCCGGTAACCAGGACTGAAC-3'	
embB	arabinosyltransferase	Ethambutol	F:5'-CATGTCATCGGCGCGAATTCG-3'	[10]
			R:5'-TGGCAGGCGCATCCACAGACT-3'	

PncA	pyrazinamidase/nicotinamidase PncA	Pyrazinamide	F:5’-GACGTATGCGGGCGTTGA-3’	[71;72]
			R:5’-CCATCAGGAGCTGCAAACCA-3’	
gyrA	DNA gyrase subunit A	Ofloxacin, Moxifloxacin	F:5’-GGTGCTCTATGAAATGTTCG-3’	[24]
			R:5’-GCTTCGGTGTACCTCATCG-3’	
gyrB	DNA gyrase subunit B		F:5’-CGATGTTCCAGGCGATACTT-3’	[24]
			R:5’-ATCTTGTGGTAGCGCAGCTT-3’	
rrs	16S ribosomal RNA	Kanamycin, Amikacin	F:5’-GTAATCGCAGATCAGCAACG-3’	[24;10]
			R:5’-TTTTCGTGGTGCTCCTTAGAA-3’	
eis	enhanced intracellular survival protein	Amikacin, Kanamycin	F:5’-AAATTCGTCGCTGATTCTCG-3’	[24]
			R:5’-CGCGACGAAACTGAGACC-3’	
rpsL or rrs	30S ribosomal protein S12/16S ribosomal RNA	Streptomycin	F:5’-GCGCCCAAGATAGAAAG-3’	[10]

			R:5’-CAACTGCGATCCGTAGA-3’	
ddn	deazaflavin-dependent nitroreductase	Delamanid	F:5’-CGAGCGCACCGACCAGAGC-3’	[63]
			R:5’-GCATGGCCCGCAGGTGGACAA-3’	
fbiA	2-phospho-L-lactate transferase		F:5’-GCGGTTCTGTTGTGGTTGGG-3’	[63]
			R:5’-CCGATGACGGGCAGGATCTCGATGG-3’	
fgd1	F420-dependent glucose-6-phosphate		F:5’-CGTGGCCGCGAGCGAGGTGAA-3’	
			R:5’-CGCCCGAACCGTCAACAACACTGG-3’	
Rv0678	hypothetical protein	Bedaquiline	F:5’-GTATCCAGGCACGCTTGA-3’	[63]
			R:5’-CCCCACAATCGATAACC-3’	
atpE	ATP synthase subunit C		F:5’-GTACTTCAGCCAAGCGATGG-3’	[63]
			R:5’-CCGTTGGGAATGAGGAAGTTG-3’	

ethA	monooxygenase EthA	Ethionamide	F:5′-CCTGGCAGCTTACTACGTGTC-3	[75]
			R:5′-CGGCATCATCGTCGTCTG-3′	
ethR	HTH-type transcriptional repressor		F:5′-TTTTCCAGGATGGCGTAGC-3′	[75]
			R:5′-CCGACCGGATCGTCAACA-3′	
alr	alanine racemase	Cycloserine	F:5’-GAAAATAAAAGACACGCCTACTTTCGCTCCA-3’	[70]
			R:5’-GACATCCATCGCCATGGCAATACCCTT-3’	

Table S2: Median (±SD) Log10 concentrations of the antimicrobial resistance genes measured in the influent and effluent wastewater.

Gene	WWTP A			WWTP B			WWTP C		
	**Influent**	**Effluent**	**Mean log reduction**	**Influent**	**Effluent**	**Mean log reduction**	**Influent**	**Effluent**	**Mean log reduction**
katG	2.36(±0.07)	2.43(±0.15)	-0.06(±0.21)	2.62(±0.19)	2.61(±0.30)	0.01(±0.12)	2.38(±0.12)	2.36(±0.08)	0.02(±0.20)
rpoB	3.00(±0.20)	2.11(±0.18)	0.90(±0.37)	3.83(±0.04)	2.36(±0.18)	1.46(±0.15)	2.17(±0.08)	1.55(±0.43)	0.62(±0.36)
embB	3.78(±0.10)	3.05(±0.08)	0.73(±0.16)	3.34(±0.10)	3.11(±0.15)	0.23(±0.17)	3.74(±0.04)	3.14(±0.06)	0.60(±0.04)
pncA	2.16(±0.06)	1.86(±0.09)	0.30(±0.15)	2.04(±0.20)	2.00(±0.19)	0.04(±0.20)	1.90(±0.26)	2.09(±0.01)	-0.19(±0.26)
rrs	5.18(±0.01)	4.37(±0.02)	0.81(±0.01)	4.71(±0.06)	4.58(±0.01)	0.14(±0.07)	4.73(±0.01)	4.67(±0.06)	0.07(±0.05)
gyrA	2.95(±0.05)	2.08(±0.05)	0.87(±0.10)	2.94(±0.09)	2.53(±0.13)	0.42(±0.11)	3.23(±0.25)	2.75(±0.11)	0.47(±0.23)
gyrB	4.51(±0.04)	4.01(±0.07)	0.50(±0.10)	1.82(±0.11)	3.31(±1.09)	-1.49(±1.15)	4.26(±0.03)	4.07(±0.03)	0.19(±0.02)
atpE	4.21(±0.07)	3.27(±0.03)	0.94(±0.09)	3.41(±0.96)	3.68(±0.07)	-0.27(±0.89)	4.05(±0.08)	3.51(±0.15)	0.54(±0.16)
ethR	3.40(±0.06)	3.56(±0.09)	-0.16(±0.15)	3.56(±0.06)	3.91(±0.06)	-0.35(±0.03)	3.67(±0.03)	4.14(±0.01)	-0.47(±0.04)
eis	4.30(±0.09)	3.65(±0.09)	0.65(±0.18)	4.13(±0.13)	4.01(±0.09)	0.12(±0.05)	3.88(±0.07)	4.07(±0.09)	-0.19(±0.07)

WWTP A

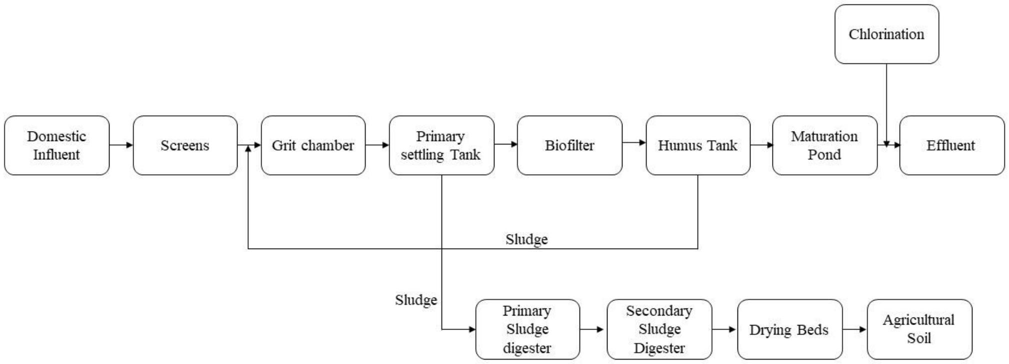

Figure S1: A schematic diagram of the Isipingo wastewater treatment plant in Durban, South Africa

WWTP B

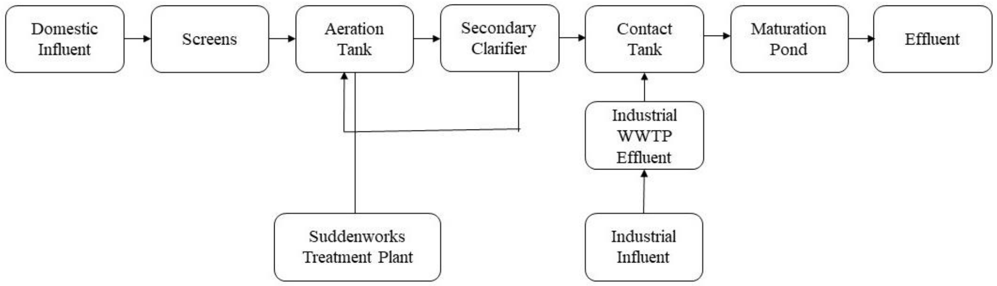

Figure S2: A schematic diagram of the Shallcross (also known as UMhlathuzana) wastewater treatment plant in Durban, South Africa

WWTP C

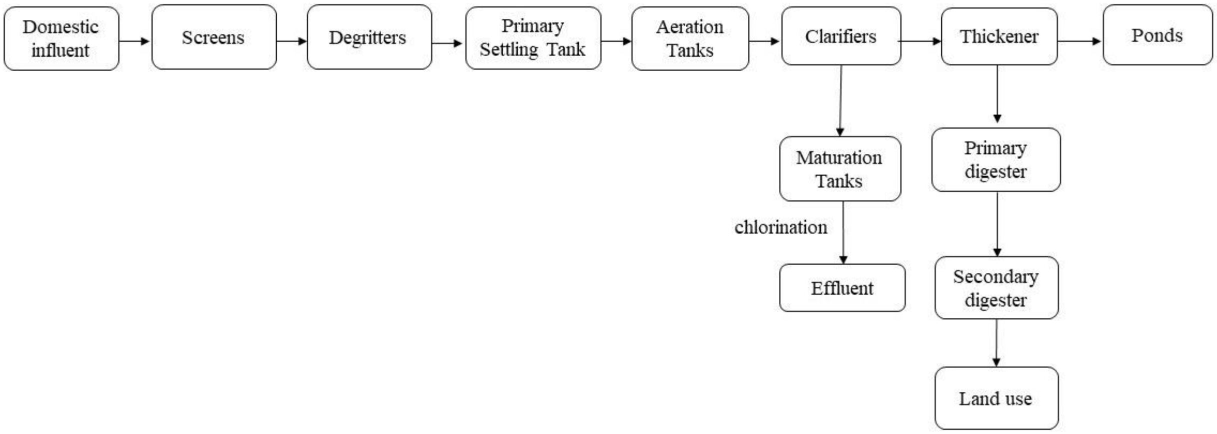

Figure S3: A schematic diagram of the Northern works wastewater treatment plant in Durban, South Africa
